# Prevalence of carriage of ESBL producing enterobacteriaceae in a Dutch teaching hospital

**DOI:** 10.1186/1753-6561-5-S6-P133

**Published:** 2011-06-29

**Authors:** G Moen, I Willemsen, M Kluytmans, C Verhulst, J Kluytmans

**Affiliations:** 1 Amphia Ziekenhius, Breda, Netherlands

## Introduction / objectives

The prevalence of Extended Spectrum Beta-Lactamase producing Enterobacteriaceae (ESBL-E) is rapidly increasing worldwide. This study aimed to determine the prevalence of ESBL-E carriage in hospitalised patients and the relative contribution of nosocomial acquisition.

## Methods

In November 2010, a prevalence survey was performed in all patients that were hospitalised in a Dutch teaching hospital by taking rectal swabs. Swabs were placed in a selective broth and incubated overnight. A subculture on a selective agar (EbSA,Cepheid) was performed. Species identification and susceptiblity testing was performed for all isolates that grew on the EbSA agar using Vitek 2 (bioMérieux). For ESBL-suspected isolates (MIC ceftazidime and/or cefotaxime >1 ug/ml) the presence of ESBL was confirmed using the combined disk diffusion method for cefotaxime and ceftazidime, both with and without clavulanic acid (Rosco). Raman Spectroscopy (SpectraCell RA) was used to determine the similarity between the ESBL-E isolates.

## Results

Rectal swabs were obtained from 559 of 668 (84%) hospitalised patients. The prevalence of ESBL-E carriage was 4,5 % (25/559). *E. coli* was the predominant species (96%). Age, sex, stay on the ICU, and length of hospital stay were not statistical significantly associated with ESBL-E carriage. Raman Spectroscopy revealed that 88% (22/25) of ESBL-E were unique isolates. One cluster of three patients was identified, and two of these patients could be epidemiologically linked.

## Conclusion

In conclusion, the observed prevalence of ESBL-E carriage of 4.5% in hospitalised patients is unexpectedly high considering the previously reported ESBL-E rates in the Netherlands. Only one case of nosocomial transmission was found, indicating the presence of an extensive and diverse reservoir of ESBL-E in the community.

## Disclosure of interest

None declared.

